# Evaluation of virtual non-contrast detector-based spectral CT images in comparison to true unenhanced images in 20 rabbits

**DOI:** 10.3389/fvets.2025.1521986

**Published:** 2025-03-13

**Authors:** Manon Mikić, Philipp Lietz, Julie-Ann Dierig, Sebastian Meller, Michael Pees, Kristina Merhof

**Affiliations:** ^1^Clinic for Small Animals, University of Veterinary Medicine, Hannover, Germany; ^2^Department of Small Mammal, Reptile and Avian Medicine and Surgery, University of Veterinary Medicine, Hannover, Germany

**Keywords:** virtual non-contrast (VNC), computed tomography, rabbits, detector-based spectral CT, true unenhanced images

## Abstract

**Introduction:**

Spectral detector Computed Tomography (SDCT) enables generation of virtual non-contrast (VNC) images derived from a post-contrast scan, as previously investigated in healthy dogs. This technique is especially promising for awake scanned rabbits where motion between the pre- and the post-contrast scans makes comparison challenging. This study aimed to determine the reliability of VNC images for assessing abdominal organs in 20 rabbits by evaluating their qualitative and quantitative parameters compared to true unenhanced (TUE) images. Our hypothesis were that the VNC series would be comparable to TUE series and that the quality of the VNC images would be equal to or even superior to the native images.

**Methods:**

Attenuation values of VNC and TUE series were assessed using a two one-sided t-test (TOST) and the signal-to-noise ratio was calculated for each ROI in the different series. Additionally, a qualitative assessment of the VNC images relative to TUE images was performed in consensus by a board-certified radiologist and a second year diagnostic imaging resident by evaluating the iodine subtraction, image quality and noise of VNC images based on a 5-point Likert scale.

**Results:**

A total of 219 regions of interest (ROIs) where drawn in abdominal organs. 72.1% of the ROIs displayed differences of less than 15 Hounsfield Units (HU) between TUE and VNC images. The differences in attenuation values of TUE and VNC were statistically significant (*p* < 0.05) for ≤ 5 HU in the spleen, for ≤ 10 HU and ≤ 15 HU additionally in the liver, musculature and renal cortices. These findings support the equivalence between VNC and TUE images. An average score of 4.4 was achieved for iodine subtraction across all patients, which encompasses all organ values, with no individual patient scoring below 4.0.

**Discussion:**

VNC images present a promising alternative to TUE images for abdominal organs without pathology in rabbits with the benefit of eliminating motion between the compared series and reducing examination time and radiation exposure by replacing pre-contrast scans. Further research is necessary to demonstrate the applicability of the technique to morphologically diseased organs.

## 1 Introduction

In recent years, a significant increase in the number of pet rabbits in European households has been noted (1). In the United Kingdom, rabbits constitute approximately 2% of the total pet population, including dogs, cats, rabbits and other species (2). Correspondingly, there has been a noted rise in the frequency of veterinary visits and the amount of diagnostic procedures performed (3). The use of computed tomography (CT) of various diseases for these patients is becoming the standard of care due to the ability to produce multiplanar reconstructions, to evaluate structures without superimposition and due to the high spatial resolution (1, 3–9). The scans can be performed while the patient is conscious which is especially interesting for rabbits as their anesthetic risk is higher compared to our canine patients (10). However, due to the low soft tissue contrast of true unenhanced images (TUE), iodinated contrast medium is frequently administered and offers a higher diagnostic value (11, 12). The use of intravenous iodinated contrast medium is well-tolerated and safe in rabbits (13). Potential movement during the scans can lead to streak artefacts but movement between the pre- and post-contrast scans often poses an additional challenge, potentially limiting the comparability of the scans (14). Dual-energy CT (DECT) has the ability to differentiate materials of different effective atomic numbers with comparable Hounsfield units (HUs) in conventional CT studies by a separate assessment of low and high-energy attenuation profiles (11, 15). Technically, this concept can be achieved in different ways. In the case of detector-based spectral DECT technology, two different layers of scintillator detectors are employed. The upper layer of the detector exhibits absorptive properties regarding low-energy photons while exhibiting permeability to high-energy photons, which are absorbed by the lower layer. This results in the generation of low- and high-energy images, and through weighted summation, full-spectrum images are calculated. The spectral data are automatically generated with each scan without the need to modify the scan parameters (16). With the aid of post-processing algorithms, iodine-containing voxels can be identified to create virtual non-contrast (VNC) images. In case the quality of VNC images proves to be sufficient in the future, pre-contrast scans could potentially be omitted (12, 15). The limitations caused by motion artefacts between the pre and post-contrast scan could thus be eliminated and the radiation dose and examination time for patients could be reduced by 50% due to the absence of the pre-contrast scan. Various studies in human medicine (11, 12, 16–18), as well as one veterinary study (19), have demonstrated good to excellent comparability between VNC and TUE images. These studies employed various comparison methods, including subjective assessments of the series and objective analyses of spectral detector computed tomography (SDCT) quality parameters such as signal-to-noise ratio (SNR), contrast-to-noise ratio, edge sharpness, and equivalence of Hounsfield Units (HUs) of identical regions of interest (ROIs) in congruent image material. The authors are not aware of any studies that have applied this technique to rabbits.

The objective of this study therefore was to assess the comparability of TUE and VNC images based on measurements in various abdominal organs and to assess the reliability of VNC images. We hypothesized that (a) the calculated VNC series would not significantly differ from the TUE series, and (b) that the quality of the VNC images would be equal or superior to that of the TUE images.

## 2 Materials and methods

This was a retrospective, single-institutional study. Institutional and medical image archives were searched from March 2023 to April 2024 for rabbits that had undergone a full body or at least abdominal CT study at the Clinic for Small Animals of the University of Veterinary Medicine Hannover, Germany, using a spectral dual-energy CT scanner (Philips IQon Spectral CT, Philips Healthcare Germany). Rabbits were included in the study if (a) a standardized SDCT protocol with complete pre-and post-contrast scans of the patients were obtained, (b) the CT study was of diagnostic quality and (c) a complete signalment including age, sex, neuter status, body weight, and reason for presentation was available. All patients were scanned consciously and placed into a device designed to minimize movement. Patients with incomplete abdominal CT scans, lacking post-contrast studies or severe motion artefacts were excluded. Additionally, patients with pathological imaging findings in the liver, spleen, kidneys, paraspinal musculature, aorta, caudal vena cava, abdominal fat, or within the urinary bladder were excluded. Patients were also excluded if a corresponding region of interest (ROI) with a minimum size of five square millimetres could not be drawn. All cases were collected by a second-year resident of diagnostic imaging (M.M.) and confirmed in consensus with a board-certified veterinary radiologist (K.M.).

### 2.1 SDCT protocol

All examinations included an initial true unenhanced (TUE) series, followed by a venous phase with the conscious patient positioned head first in prone recumbency. SDCT scans were acquired using an automated adapted tube current, a tube potential of 120–140 kV, a pitch of 0.4, a gantry rotation time of 1 s, a slice thickness of 0.9 mm, and a 512 × 512 image matrix. The volume computed tomography dose index (CTDI_vol_) of the scans ranged between 36.6 and 51.9 mGy, while the scan size of the rabbits varied from 11.2 to 26.4 cm. Scans were performed following a standardized abdominal protocol, employing both soft tissue (HB) and bone kernels (YC) with appropriate bone window settings (window length: 800; window width: 2000) and soft tissue window settings (window length: 60; window width: 350). Post-contrast CT scans were obtained approximately 60 s after the manual intravenous administration of non-ionic contrast media into the auricular vein. Each patient received 700 mg iopamidol/kg bodyweight [2.3 mL/kg of Solutrast^®^300, Bracco Imaging Deutschland GmbH, Konstanz, Germany].

### 2.2 Quantitative image analysis

**Figure 1:**
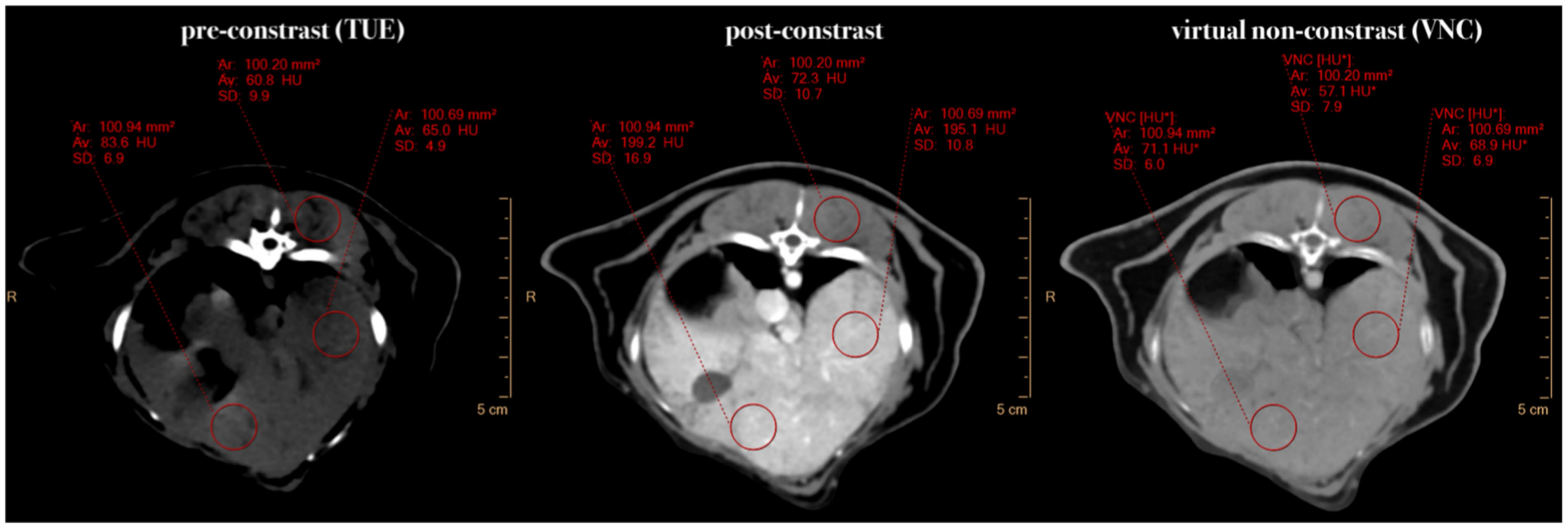
Comparative presentation of synchronized pre-contrast (TUE), post-contrast, and virtual non-contrast (VNC) images in the transverse plane. If it was not possible to draw a ROI of 100mm^2^, the ROI size was chosen to be “as large as possible.” Note the HU differences of ≤ 15 HU in the liver and ≤ 10 HU in the musculature between TUE and VNC images.

All images were analyzed by a second-year diagnostic imaging resident (M.M.) on a computer workstation equipped with system-associated software (IntelliSpace Portal Version 11.x/Philips Healthcare) and a calibrated flat-panel Retina display monitor (HP Deutschland GmbH, Boeblingen, Germany) certified for image analysis. In the first step, VNC images were reconstructed from the provided spectral data using system-specific software. The image series of conventional TUE, conventional post-contrast and VNC images of the abdomen were then linked and synchronized to allow for an exact comparison of the sections. In cases where synchronization of the series failed due to patient movement between scans, manual adjustment of the image pairing was performed. The images were examined exclusively in a soft tissue window (window level: 60; window width: 350). Adjustments to the windowing were permitted for the reviewer. In the subsequent phase of the process, multiple circular regions of interest were drawn in the TUE images in seven different organs. Wherever feasible, ROIs were sized at 100 mm^2^ ± 2 mm^2^. If this size was not applicable to an organ to avoid including extraneous structures (such as in the spleen, aorta and renal cortex), the largest possible circular ROI was selected. The ROIs needed to have a minimum of five square millimetres. The ROIs were then transferred to the paired post-contrast and VNC images using copy/paste, ensuring that both the location and size of the ROIs were identical (Figure 1). The following locations were selected for analysis:

1. Liver (2 ROIs, avoiding larger vessels: in right and left liver lobes).
2. Spleen (2 ROIs).
3. Renal cortex bilaterally (1 ROI each).
4. Abdominal aorta (1 ROI) at the level of the diaphragm.
5. Paravertebral muscles (1 ROI each right and left, at the level of the transition between the 13th thoracic and first lumbar vertebrae).
6. Intra-abdominal fat (1 ROI).
7. Urinary bladder (1 ROI).

The different average HUs were documented for each paired ROI measurement in the VNC and TUE images, whereby the HUs of the ROIs were automatically averaged. Based on a categorization derived from medical studies (11, 16, 17) and a preceding study by the authors (19), the data were classified into four distinct groups.

1. Difference between VNC_HU_ and TUE_HU_ = ≤ 5 HU
2. Difference between VNC_HU_ and TUE_HU_ = ≤ 10 HU
3. Difference between VNC_HU_ and TUE_HU_ = ≤ 15 HU
4. Difference between VNC_HU_ and TUE_HU_ = > 15 HU

### 2.3 Qualitative image analysis

The qualitative analysis included the assessment of image quality and noise, as well as the efficiency of iodine subtraction. The analysis was performed in consensus by a board-certified veterinary radiologist (K.M.) and a second-year diagnostic imaging resident (M.M.). Image quality was assessed by comparing the overall appearance of the gastrointestinal tract, hepatic and splenic parenchyma, kidneys, paraspinal musculature, urinary bladder and major vessels (aorta and caudal vena cava) in paired TUE and VNC images using a 5-point Likert scale (Table 1).

**Table 1:** Modified five-point-likert-scale for assessment of image noise and quality in VNC compared to TUE images.

Image noise and quality: VNC vs. TUE images	
1	VNC markedly worse than TUE images
2	VNC mildly worse than TUE images
3	VNC equivalent to TUE images
4	VNC mildly better than TUE images
5	VNC markedly better than TUE images

The efficiency of iodine subtraction in VNC images was evaluated by direct comparison with the paired conventional pre- and post-contrast images using a modified 5-point Likert scale (Table 2).

**Table 2:** Modified five-point-Likert-scale for assessing the iodine subtraction from VNC images in comparison with TUE images.

Iodine subtraction in VNC images	
1	Insufficient subtraction of iodine
2	Partly sufficient subtraction of iodine with larger, incomplete areas in the parenchyma
3	Moderately sufficient subtraction of iodine with incomplete areas in parts of the parenchyma
4	Almost complete subtraction of iodine
5	Complete subtraction of iodine

### 2.4 Statistical methods

Dedicated software was used for statistical data evaluation and visualization (Microsoft® Excel, GraphPad Prism 10). VNC and TUE images of each organ or tissue were grouped and compared by subtracting the HU of every ROI in VNC from the corresponding ROI in the TUE images. If multiple ROIs per organ were used, the differences between values in VNC and TUE images were averaged. The equivalence between the VNC and TUE series was tested using a two-one-sided t-test (TOST) with an opposing null hypothesis. This null hypothesis assumed a difference between VNC and TUE images, with deviation limits of ≤5, ≤10, or ≤ 15 HUs. If this hypothesis was significantly rejected (*p* ≤ 0.05), equivalence between both techniques within the specific deviation limit was assumed, thereby confirming the original study hypothesis. The distribution of animals with HU differences within the corresponding limits was also calculated for each organ system or tissue as a percentage. Additionally, a qualitative analysis of the VNC and TUE data was conducted by documenting the mean values for each patient and individual organs by two observers (M.M. and K.M.) in consensus, as described in the section of qualitative analysis. To investigate relationships between the weight of animals and the differences between TUE and VNC values, Pearson correlations were applied for each organ system or tissue. Tests were two-sided and a *p*-value of ≤0.05 was considered significant. To investigate the normal distribution of the dara, a Shapiro–Wilk test was performed.

## 3 Results

### 3.1 Study population

A total of 20 CT scans met the inclusion criteria, based on a total of 87 abdominal or full-body CT scans from client-owned rabbits which were identified and reviewed during the study period from February 2023 to April 2024. As post-contrast scans were traditionally not performed as a standard procedure in our institution by the time the data was collected, 66 studies had to be excluded. One additional study was excluded due to excessive motion artefacts, which made a comparison of the pre- and post-contrast scans impossible. The presenting complaint of the patients included one or more of the following symptoms: inappetence, apathy, chronic or acute weight loss, respiratory distress, hypothermia, polyuria, stanguria, trauma, ocular discharge, previously by ultrasound diagnosed nephrolithiasis, precardial mass, liver cysts or cardiomyopathy. The most commonly reported clinical complaint was weight loss despite normal food intake. The study population included 10 female and 10 male rabbits, with 9 (45%) neutered males, 1 (5%) sexually intact male, 6 (30%) female neutered and 4 (20%) sexually intact females. The average age was 5.8 years, with a range from 1 to 11 years (SD: ± 2.56). The rabbits ranged from 1.27 kg to 6.48 kg in body weight (mean and SD: 2.81 ± 1.61).

### 3.2 Quantitative analysis

A total of 219 ROIs were drawn. In one patient, the right kidney was absent due to a prior nephrectomy, so only the cortex of the left kidney could be measured in this case. The difference between TUE and VNC attenuation values was ≤ 15 HU in 72.1%, ≤ 10 HU in 60% and ≤ 5 HU in 30.7% of all compared ROIs. All measured ROIs of each organ or tissue were found to be normally distributed and averaged ROIs showed a Gaussian distribution according to the Shapiro–Wilk test.

The differences in attenuation values of TUE and VNC were statistically significant (*p* < 0.05) for ≤ 5 HU in the spleen, for ≤ 10 HU and ≤ 15 HU additionally in the liver, musculature and renal cortices. No statistically significant differences in attenuation values ≤ 15 HU were detected in the aorta, urinary bladder and intraabdominal fat. Table 3 summarizes the TOST results.

**Table 3:** Two one-sided *t*-test (TOST) for assessment of equivalence between VNC and TUE images (*p*-values).

Region of interest (ROI)	≤ 5 HU	≤ 10 HU	≤ 15 HU
Liver (mean out of two measurements); *n* = 40	0.1212 (*n* = 20)	**0.0006** (*n* = 32)	**<0.0001** (*n* = 34)
Spleen (mean out of two measurements); *n* = 40	**0.038** (*n* = 20)	**0.0001** (*n* = 30)	**<0.0001** (*n* = 36)
Musculature (mean out of two measurements – left and right); *n* = 40	0.1029 (*n* = 14)	**<0.0001** (*n* = 38)	**<0.0001** (*n* = 40)
Renal cortex (mean out of two measurements – left and right); *n* = 39	0.7005 (*n* = 8)	**0.0498** (*n* = 24)	**0.0004** (*n* = 30)
Aorta; *n* = 20	0.9979 (*n* = 6)	0.9367 (*n* = 9)	0.4765 (*n* = 11)
Urinary Bladder; *n* = 20	0.9931 (*n* = 6)	0.9953 (*n* = 9)	0.802 (*n* = 13)
Intraabdominal Fat; *n* = 20	1 (*n* = 0)	0.9998 (*n* = 4)	0.9757 (*n* = 7)

A *p*-value ≤ 0.05 shows equivalence within the specified HU-delta (tolerance limits ≤5 , ≤10,≤15 HU difference of VNC towards TUE). Significant values are marked in bold. *n* = number of ROIs.

The smallest differences in HUs between TUE and VNC images were observed in the liver, spleen, and paraspinal musculature. In these cases, 80, 75, and 95% of the measurements, respectively, fell within the threshold of ≤ 10 HU difference. The most notable discrepancy in the HUs between VNC and TUE was observed in the assessments of intra-abdominal fat and the aorta. Consequently, only 35 and 55% of the measurements were within the acceptable range of ≤ 15 HU. It is important to note that these results are not statistically significant as shown in Table 3. As shown in Table 3, it is also evident that for liver, spleen, and muscle measurements, only 6, 4, and none of the 40 measured ROIs, respectively, exceeded the threshold of 15 HUs. In contrast, 10 out of 40 ROIs in the renal cortex, 7 out of 20 in the urinary bladder, 9 out of 20 in the aorta, and even more than half of the measurements (13) in intra-abdominal fat were above this threshold.

**Figure 2:**
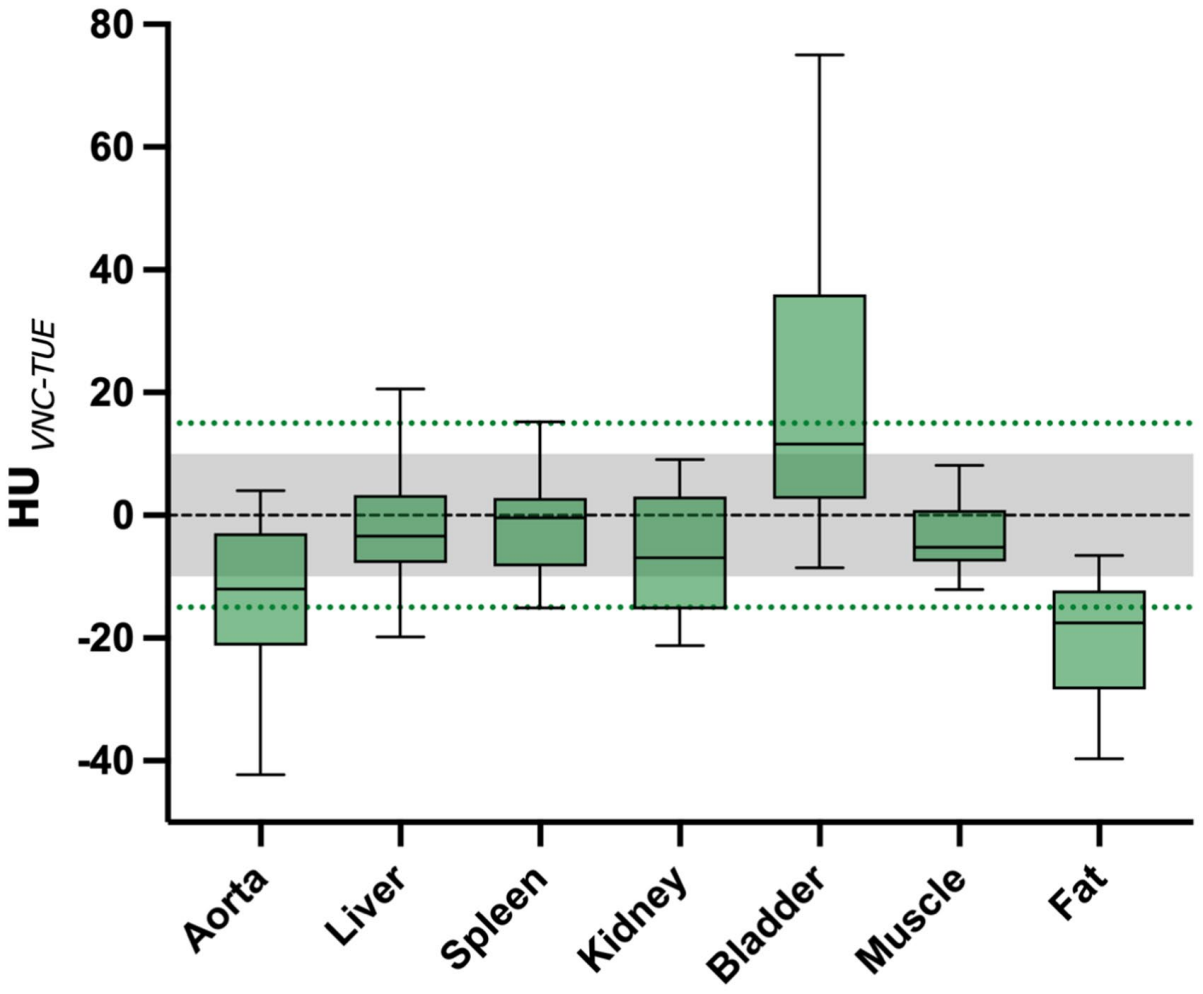
The mean difference between VNC and TUE for all ROIs is illustrated. The threshold of 10 HU difference, which is considered “negligible,” is indicated by the grey shaded area, while the threshold of 15 HU difference, which is considered “acceptable,” is represented by the green dashed line. The lower and upper margins of each box represent the 25th and 75th percentiles, respectively. The median is marked by a black line within the plots.

The average HU value difference between TUE and VNC was ≤10 HU in the majority of measurements in the liver, muscle, and spleen, and ≤ 15 HU in the renal cortex. For all regions, the values are summarized and displayed with the range of the standard deviation using a box-and-whisker plot in Figure 2.

Correlations between the weight of animals and the distances of the difference between VNC and TUE images to 0 were negative in each organ system or tissue except for the bladder. A significant correlation (r = −0.6723; *p* = 0.0012) with a moderate fit (R^2^ = 0.5) was found only for the kidneys.

### 3.3 Qualitative analysis

An average score of 4.4 was achieved for iodine subtraction across all patients, which encompasses all organ values, with no individual patient scoring below 4.0. This indicates that there has been a nearly complete subtraction of iodine on average. However, smaller intraparenchymal vessels were in general not sufficiently suppressed and therefore not included in the assessment. Scores of at least 4.6 were achieved for the liver, spleen, caudal vena cava, aorta, and musculature. Iodine subtraction values for the bladder, gastrointestinal tract, and renal cortices were at least 3.8, 3.9, and 3.5, respectively. The detailed values from this evaluation are presented in Table 4.

**Table 4:** Iodine subtraction score for 20 rabbits.

Location	Rabbits																				Average score location
	1	2	3	4	5	6	7	8	9	10	11	12	13	14	15	16	17	18	19	20	
Liver	5	5	4	5	5	3	5	5	4	5	5	5	4	5	3	4	5	5	5	5	4.6
Spleen	5	5	5	5	5	5	5	5	5	5	5	5	5	5	5	5	5	5	5	5	5.0
Kidneys	4	4	3	5	4	3	4	3	3	4	4	4	3	3	3	3	4	4	3	2	3.5
GIT	3	4	3	5	4	4	4	3	3	5	5	4	4	4	3	4	4	4	4	4	3.9
CVC	4	5	5	5	5	5	4	4	5	5	5	5	5	5	5	5	5	4	4	5	4.8
Aorta	5	5	5	5	5	5	5	5	5	5	5	5	5	5	5	5	4	5	4	5	4.9
Urinary bladder	5	4	4	4	4	2	5	4	5	3	5	4	4	2	3	3	3	4	5	2	3.8
Musculature	5	5	5	5	5	5	5	5	5	5	5	5	5	5	5	5	5	5	5	5	5.0
Average score patient	4.5	4.6	4.3	4.9	4.6	4.0	4.6	4.3	4.4	4.6	4.9	4.6	4.4	4.3	4.0	4.3	4.4	4.5	4.4	4.1	4.4

Note the complete iodine subtraction in spleen and musculature and almost complete subtraction in aorta, caudal vena cava (CVC) and liver. The iodine subtraction score for the gastrointestinal tract (GIT), urinary bladder and kidneys is still ≥3.5 . 1: insufficient subtraction of iodine, 2: Partly sufficient subtraction of iodine with larger, incomplete areas in the parenchyma, 3: Moderately sufficient subtraction of iodine with incomplete areas in parts of the parenchyma, 4: almost complete subtraction of iodine, 5: complete subtraction of iodine.

The mean image quality score was 4.2 on the 5-point Likert scale. A single patient was assigned a rating of “3” on the five-point Likert scale, which represents the lowest rating in the study.

## 4 Discussion

The calculated VNC values for the liver, spleen, renal cortices, and musculature were equivalent to the measurements in the TUE series which confirmed our first hypothesis.

Under favourable conditions, human observers can distinguish between 700 and 900 shades of grey displayed simultaneously across the available brightness range of the medical display. The human eye is capable of detecting only a 6% change in grayscale (20). In practical terms, this means that in a reconstructed soft tissue window with a greyscale range of 290 Hounsfield Units, a discrimination of a difference of about 17 HUs is possible for the human eye.

Based on this, previous studies have established a negligible threshold of ≤ 10 HU between VNC and TUE images. Additionally, an acceptable threshold of ≤ 15 HUs has been established (16, 17).

The highest discrepancies in HU values were observed in the aorta, urinary bladder and intra-abdominal fat. This finding is consistent with those of previous studies. It should be noted that, in contrast to the liver, musculature, spleen, and renal cortex, which exhibited averaged ROI values, these organs displayed only a single ROI, which could have increased the measurement bias. Sauter et al. (16) sought to elucidate this phenomenon by postulating that the exceedingly high iodine concentrations within the aorta might lead to disproportionately elevated iodine subtraction rates through the algorithm. Another study attributed the incomplete subtraction of motion artefacts of the aorta, which resulted in a minimal spatial difference between the two tubes and, consequently, an impact on the output of the VNC algorithm (21).

**Figure 3:**
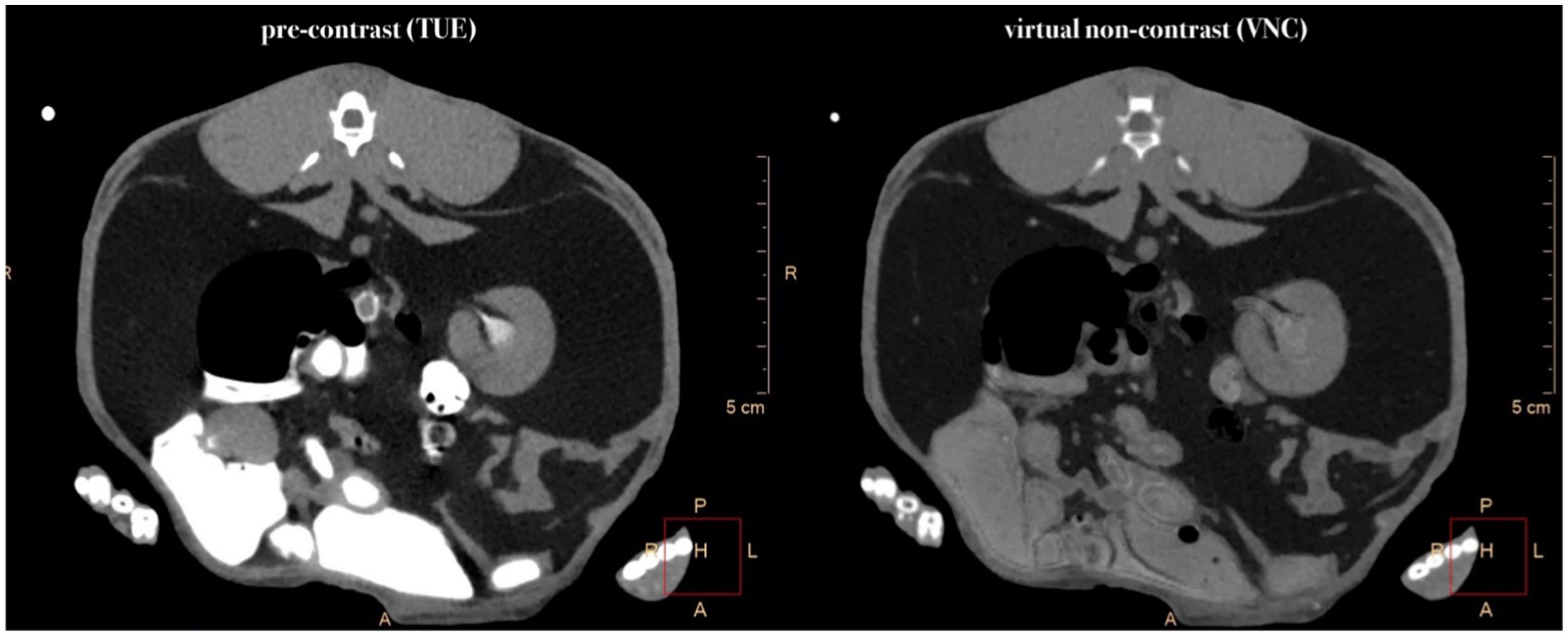
Comparative presentation of synchronized pre-contrast (TUE) and virtual non-contrast (VNC) images in the transverse plane. Note the partly subtraction of iodine in the region of the renal pevis and the almost complete subtraction of mineral-attenuating structures within the intestinal tract.

Rabbits are distinctive in their calcium utilization, exhibiting a markedly elevated absorption rate from the digestive tract. This hyperabsorption of dietary calcium can result in excessive calcium excretion in the urine, leading to the formation of a typical sludge in the bladder (22). The distinction between contrast medium and calcium or calcified tissue depends on the CT density of the tissues and is limited when the CT attenuation is low (23). Suppression of iodine-like attenuation might interfere with calcium-like attenuation (24) which may result in normal calcium or calcium sludge within the bladder and renal parenchyma being subtracted, thereby limiting the comparability of TUE and VNC images in this region (Figure 3). This observation was also partially noted in the renal cortices, where smaller mineralizations were sometimes removed by the VNC algorithm during the study. If this pitfall poses a source of relevant errors in clinical interpretation in diseased patients needs to be evaluated in future studies.

In addition, the calculation of the VNC images is based on the measurement of water and iodine, which are in turn based on the photoelectric effect and Compton scattering. In contrast to the parenchymal organs, the intraabdominal fat is not situated on the water line as a reference point, but rather beneath it (19). This may explain the reduced accuracy of the algorithm in adipose tissue. If this weak point of the algorithm has a clinically relevant impact on certain pathologies like for example the fatty infiltration of the liver, needs to be evaluated in further studies.

The second hypothesis regarding the superior image quality of VNC images compared to the conventional TUE images was also confirmed. Similar improvements in image quality with VNC images have been demonstrated in both human medical studies (16, 17) and a veterinary study (19). In 72.1% of the measured ROIs, a discrepancy of ≤ 15 HUs between VNC and TUE images was observed. A difference of ≤15HUs was deemed acceptable given that the human eye is only capable of discerning differences of approximately 17 HUs within a specified soft tissue window with a grayscale range of approximately 290 HUs (20). This result is in accordance with the findings of previous studies. Lietz et al. addressed the same question in a cohort of clinically healthy Beagle dogs, and their findings indicated that 91.61% of the measured ROIs exhibited a difference of less than or equal to 15 HUs. In human medicine studies, values of 72% (12), 98% (17), and over 90% (16) have been reported. Similarly, Laukamp et al. achieved good results in their study comparing TUE and VNC images of the liver (25). In this study, the difference between VNC and true unenhanced images was below 10 HUs in 92.3% of the comparative liver measurements. Several factors, including patient size, voxel size, beam hardening artefacts and high iodine concentrations, have been suggested as potential influences on the accuracy of iodine subtraction (17). Given that the patients included in this study were exclusively rabbits, the impact of body size is of particular interest. However, a correlation between weight and ROIs was only observed in renal measurements (r = −0.6723; *p* = 0.0012), with a moderate fit (R^2^ = 0.5). A possible explanation for this error might be the generally small size of patients included in the study.

Our study had several limitations. Firstly, due to the small number of animals in this study, the power of our statistical analysis was limited. A second limitation was the retrospective nature and the resulting selection of patients. As the patients were selected based on specific criteria from the CT studies, the study did not only include clinically healthy animals. Although the described measurements were performed in radiologically normal organs, this does not exclude pathology. We could not prove our findings by pathology or histopathology as our study included clinical patients. Further studies with histopathologically or cytologically confirmed diseased organs could confirm the reliability of the VNC algorithm and test its constraints. Another limitation was that the rabbits were scanned consciously. This occasionally resulted in movement between the native and post-contrast scans, which in turn might have led to inaccuracies in the alignment of the images for the simultaneous ROI placements, necessitating exclusion in case of significant movement or manual adjustments when minor movement was present. However, the movement was primarily affecting the head, and therefore the impact on the region of interest was minimal. Furthermore, scanning the rabbits consciously represents the normal clinical routine. In this study, the administration of contrast medium was performed manually, as the utilisation of our contrast injector was not compatible with rabbits due to the high injection pressure. Consequently, minor discrepancies were observed in the timing of the post-contrast scans, which, given the physiological characteristics of the rabbits, could have resulted in different contrast phases. Further research could investigate the influence of the contrast phase on the comparability of VNC and TUE images.

## 5 Conclusion

In conclusion, the VNC images demonstrated an acceptable discrepancy from the TUE images in 76% of the ROIs when a minimal cutoff of ≤10 HU was applied, and in 61% of the ROIs when a minimal cutoff of ≤10 HUs was utilized. The most favourable outcomes were observed in the spleen, liver, paraspinal musculature, and renal cortex. Further research is required to ascertain whether VNC algorithms can be employed in the analysis of pathologically affected organs and whether the resulting images are comparable to those obtained using TUE data. This technique has significant potential, particularly for patients undergoing conscious scans, as it substantially reduces the radiation exposure, possible motion artifacts in between the scans and the stress associated with the scan by halving the scan time.

## Data Availability

The original contributions presented in the study are included in the article/supplementary material, further inquiries can be directed to the corresponding author.
